# Punctate Hyperfluorescence as a Favorable Predictive Factor for Treatment Response Following a Switch to Brolucizumab for Patients with Aflibercept-Refractory Neovascular Age-Related Macular Degeneration

**DOI:** 10.3390/jcm14145141

**Published:** 2025-07-19

**Authors:** Hiroyuki Kamao, Katsutoshi Goto, Kenichi Mizukawa, Ryutaro Hiraki, Atsushi Miki, Shuhei Kimura

**Affiliations:** 1 Department of Ophthalmology, Kawasaki Medical School, 577 Matsushima, Kurashiki 701-0114, Japan; k_goto@med.kawasaki-m.ac.jp (K.G.); hiraki@med.kawasaki-m.ac.jp (R.H.); amiki@med.kawasaki-m.ac.jp (A.M.); kimuras@med.kawasaki-m.ac.jp (S.K.); 2 Shirai Eye Hospital, 1339 Takasecho Kamitakase, Mitoyo 767-0001, Japan; mizu-p@shirai-hosp.or.jp

**Keywords:** punctate hyperfluorescence, brolucizumab, aflibercept-refractory neovascular age-related macular degeneration

## Abstract

**Background/Objectives:** To identify the predictive biomarkers of treatment response following a switch to brolucizumab in patients with aflibercept-refractory neovascular age-related macular degeneration (nAMD). **Methods**: This retrospective study included 47 eyes of 44 patients with nAMD who were switched to brolucizumab; a two-year follow-up was completed for 37 eyes of 34 patients after the switch. The patients were classified into two groups based on the presence (fluid group) or absence (dry group) of retinal fluid at one and two years after switching, and their visual acuity, central retinal thickness, subfoveal choroidal thickness, injection interval, and dry macular rate were evaluated. **Results**: A dry macula was achieved for approximately 80% of patients at two years after the switch (*p* < 0.001), and the mean injection interval was significantly extended from 6.4 ± 1.8 weeks to 10.5 ± 2.6 weeks during the same period (*p* < 0.001). Both the mean central retinal thickness and subfoveal choroidal thickness showed a significant decrease at two years after the switch (*p* < 0.001 for both). A significantly higher proportion of patients in the Dry group exhibited punctate hyperfluorescence in the fellow eye (*p* < 0.001), and all patients in the dry group achieved a dry macula at two years. **Conclusions**: Switching to brolucizumab may be an effective treatment option for patients with aflibercept-refractory nAMD. Punctate hyperfluorescence may serve as a favorable prognostic factor following a switch to brolucizumab.

## 1. Introduction

Neovascular age-related macular degeneration (nAMD) remains a principal cause of visual impairment and blindness in developed nations. Anti-vascular endothelial growth factor (VEGF) therapies have improved visual prognoses in affected individuals. However, undertreatment poses the risk of poor visual outcomes, which necessitates ongoing follow-up visits and imposes a substantial financial burden. Aflibercept is one of the most commonly administered anti-VEGF agents and has demonstrated beneficial effects in both clinical trial populations and real-world settings [1,2,3]. Despite its effectiveness, nearly 40% of patients continue to require injections every eight weeks [2], highlighting the need for alternative treatment options for patients with aflibercept-refractory nAMD.

Brolucizumab, approved in 2019 for the treatment of nAMD, is a humanized single-chain antibody fragment with the lowest molecular weight among commercially available anti-VEGF agents [4]. This structural advantage allows for administration at higher molar concentrations, potentially leading to enhanced and more durable therapeutic effects. In two prospective, randomized phase III trials (HAWK and HARRIER), brolucizumab demonstrated non-inferior visual and superior anatomical outcomes relative to aflibercept in treatment-naïve patients with nAMD [5,6]. Furthermore, several studies have reported the efficacy of switching to intravitreal brolucizumab (IVBr) for patients with nAMD who are refractory to aflibercept [7,8].

Treatment responses to anti-VEGF therapy vary among individuals, and the identification of reliable biomarkers for predicting treatment responses is essential for optimizing personalized treatment strategies. Hemorrhage and other pathological changes often hinder the accurate evaluation of biomarkers once nAMD develops in the affected eye. Therefore, ideal predictive biomarkers should include bilateral lesions that are identifiable before disease onset, such as drusen. However, studies investigating biomarkers associated with treatment response following a switch to brolucizumab for aflibercept-refractory nAMD remain limited [9,10,11], and no studies have analyzed biomarker associations based on outcomes over two years or longer.

This study aimed to evaluate the 2-year outcomes of switching to brolucizumab in patients with aflibercept-refractory nAMD and identify predictive biomarkers of treatment response.

## 2. Materials and Methods

### 2.1. Study Design

We retrospectively reviewed the medical records of all patients with nAMD who had retinal fluid despite receiving intravitreal aflibercept (IVA) every 4–8 weeks and were subsequently switched to intravitreal brolucizumab (IVBr) at Kawasaki Medical School between July 2020 and March 2023. Retinal fluid was defined as intraretinal fluid (IRF) and/or subretinal fluid (SRF). All enrolled patients were of Japanese ethnicity. Cigarette smoking data were obtained from hospital records or patient self-reports. Patients were categorized as never smokers or ever smokers, according to the classification used in a previous report [12]. We excluded patients who had undergone laser photocoagulation or vitrectomy, as well as those with macula neovascularization as a result of high myopia (>–6 diopters), uveitis, or angioid streaks. Patients with other ocular diseases that could potentially influence the treatment outcomes of the study eye, such as branch retinal vein occlusion, diabetic retinopathy, or glaucoma, were also excluded. Genetic testing was not performed or reviewed for any patients included in this study.

### 2.2. Treatment Method and Data Collection

All the enrolled patients continued treatment with a treat-and-extend regimen after switching from aflibercept to brolucizumab. The treatment interval was adjusted in 2 to 4 week increments for extension or shortening, and it was reduced when the retinal fluid recurred or persisted, and was extended by up to a maximum of 20 weeks if no exudation was observed for six months.

All participants underwent a complete ophthalmologic examination, including measurement of best-corrected visual acuity (BCVA), indirect ophthalmoscopy, slit-lamp biomicroscopy with a noncontact lens, color fundus photography, and fundus autofluorescence (FAF) (TRC-50DX; Topcon Corporation, Tokyo, Japan), swept-source OCT (DRI OCT-1 Atlantis; Topcon Corporation, Tokyo, Japan), fluorescein angiography (FA), and indocyanine green angiography (ICGA) (HRA-2; Heidelberg Engineering GmbH, Dossenheim, Germany). Visual acuity data were obtained as decimal visual acuity (BCVA) values and converted to the logarithm of the minimum angle of resolution (logMAR) units for analysis. Central retinal thickness (CRT) and subfoveal choroidal thickness (SFCT) were measured using swept-source OCT as previously described [12]. Punctate hyperfluorescence (PH) was investigated in all fellow eyes using mid- to late-phase ICGA based on previous studies [13,14]. In this study, the early phase of FA and ICGA was defined as approximately 20–60 s after dye injection, and the late phase was defined as approximately 10–15 min after injection. PH is typically distributed along the choroidal vessels and appears as solitary or clustered hyperfluorescent spots within areas of choroidal vascular hyperpermeability on late-phase ICGA. The early phase FA and ICGA correspond to approximately 1–2 min after dye injection, while the late phase FA and ICGA refer to approximately 10–15 min after dye injection. The type of drusen in all fellow eyes was determined based on fundus color photography, OCT, FA, and ICGA according to the criteria described in a previous study [15], classified as soft drusen, subretinal drusenoid deposit (SDD), pachydrusen, or no drusen. Subretinal hyperreflective material (SHRM) in all affected eyes, defined as a hyperreflective signal above the retinal pigment epithelium on OCT images, was classified into exudation, hemorrhage, neovascular tissue, vitelliform, fibrosis, and no SHRM based on fundus color photography, FAF, OCT, OCT angiography, FA, and ICGA, as reported previously [14]. None of the eyes in the present study had neovascular tissue, vitelliform tissue, or fibrosis. At the time of classification of PH, drusen, and SHRM, all patients had unilateral macular neovascularization (MNV). In cases where MNV subsequently developed in the fellow eye and treatment was switched to IVBr, the classifications of PH and drusen were determined based on the findings of the initially affected eye.

### 2.3. Outcome Measures

The outcome measures included changes in the presence of retinal fluid, BCVA, CRT, SFCT, injection interval, and total number of injections per year. The injection interval at each time point was defined as the number of weeks between the current and previous IVBr injections. These parameters were compared at the time of switching and one and two years after switching. The patients were classified into two groups: the dry group included those who had no retinal fluid at both one and two years after switching, and the fluid group included those who had retinal fluid at either time point. The following baseline characteristics of the dry and fluid groups were compared: age, sex, smoking history, presence of IRF and SRF, presence of polypoidal lesions, presence of PH, drusen subtype, SHRM subtype, follow-up duration before switching, total number of injections before switching, injection interval before switching, and BCVA, CRT, and SFCT at baseline.

### 2.4. Statistical Analysis

Statistical analyses were performed using the JMP Pro 17 software (SAS Institute, Cary, NC, USA). Changes over time (baseline, 1 year, and 2 years) in BCVA, CRT, SFCT, and injection interval were analyzed using the Friedman test for repeated measures. When a statistically significant difference was detected, post hoc pairwise comparisons were performed using the Nemenyi test to identify specific timepoints with significant changes. Pearson’s chi-square test was used to evaluate the differences in the prevalence of retinal fluid at each time point. The Wilcoxon rank-sum test was used to compare the age, follow-up duration, total number of injections, injection interval, BCVA, CRT, and SFCT between the two study groups. Pearson’s chi-square test was used to assess the differences in sex distribution, history of smoking, presence of IRF, SRF, polypoidal lesions, PH, Drusen subtypes, and SHRM subtypes. Statistical significance was set at *p* < 0.05. Single and double asterisks (* and **) represent *p* < 0.05 and *p* < 0.001, respectively.

## 3. Results

### 3.1. Clinical Characteristics and Treatment Outcomes in the Study Population

During the study period, 47 eyes of 44 patients switched from IVA to IVBr. Ten eyes of ten patients were excluded for the following reasons: intraocular inflammation in one eye, intraocular inflammation and retinal vasculitis in two eyes, lack of ICGA data for four eyes, switching to faricimab due to insufficient treatment response in one eye, and discontinuation of hospital visits in two eyes. A total of 37 eyes of 34 patients with nAMD were included in our study. The male and female patients accounted for 22 and 15 eyes, respectively. Their mean age was 70.9 ± 7.1 years (58–91 years), with a mean age of 71.6 ± 8.3 years for male patients and 69.8 ± 5.0 years for female patients. At the initiation of anti-VEGF therapy, the prevalence of polypoidal lesions and PH was 48.6% and 45.9%, respectively. The proportions of patients with exudation, hemorrhage, and no SHRM in the affected eyes were 8.1%, 18.9%, and 73.0%, respectively. The proportions of patients with soft drusen, SDD, pachydrusen, and no drusen in the fellow eye were 29.7%, 5.4%, 13.5%, and 51.4%, respectively. At the time of switching, the prevalence of SRF and IRF was 91.9% and 16.2%, respectively. The mean BCVAs at baseline and at one and two years after switching were 0.23 ± 0.25, 0.17 ± 0.27, and 0.22 ± 0.36, respectively. The mean BCVA at one year after switching was lower than that at baseline (*p* = 0.038), whereas there was no significant difference between the baseline and two years after switching (*p* = 0.63). The mean CRT was 240.4 ± 56.0 µm at baseline, decreasing to 197.5 ± 41.0 µm at one year and 201.9 ± 43.5 µm at two years after switching (one year, *p* < 0.001; two years, *p* < 0.001). The mean SFCTs at baseline and at one and two years after switching were 193.2 ± 101.4, 179.8 ± 101.4, and 163.7 ± 97.9 µm, respectively, indicating a progressive reduction over the study duration (baseline vs. one year, *p* < 0.01; baseline vs. two years, *p* < 0.001; one year vs. two years, *p* < 0.01). The mean injection interval at baseline and at one and two years after switching were 6.4 ± 1.8, 9.0 ± 1.5, and 10.5 ± 2.6 weeks, respectively, showing a gradual extended over time (baseline vs. one year, *p* < 0.001; baseline vs. two years, *p* < 0.001; one year vs. two years, *p* < 0.01). The mean number of IVBr injections was 6.8 ± 0.9 during the first year and 5.3 ± 1.2 during the second year after switching.

### 3.2. Treatment Outcomes of the Dry and Fluid Groups

**Figure 1:**
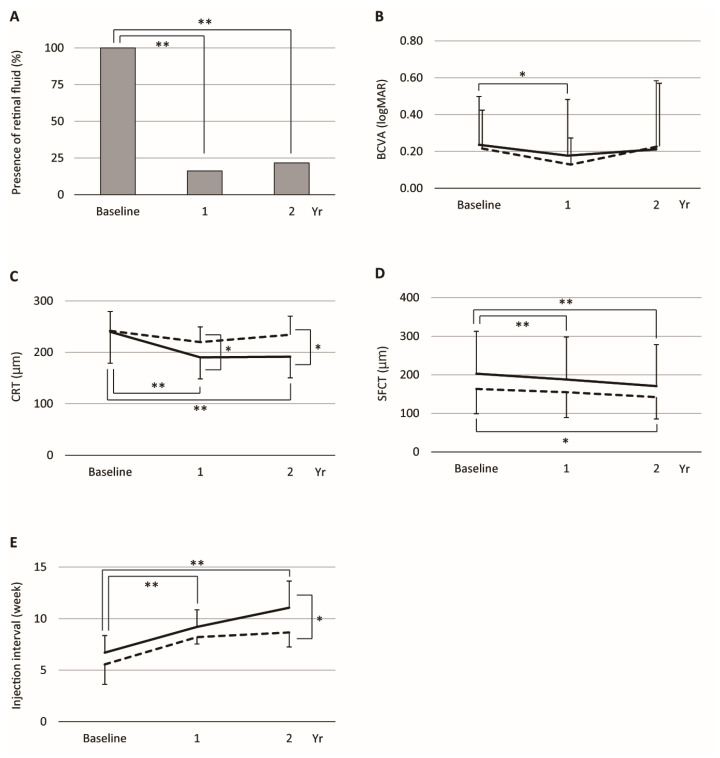
Treatment outcomes of the dry and fluid groups. (**A**) The presence of retinal fluid, defined as IRF and/or SRF, at the baseline, 1 year, and 2 years. (**B**) BCVA (logMAR) at the baseline, 1 year, and 2 years after switching. (**C**) CRT (µm) at the baseline, 1 year, and 2 years. (**D**) SFCT (µm) at the baseline, 1 year, and 2 years. (**E**) Injection interval (weeks) between IVBr injections at each time point. *, *p* < 0.05; **, *p* < 0.001. Solid lines represent the dry group, and dashed lines represent the fluid group. BCVA, best-corrected visual acuity; CRT, central retinal thickness; SFCT, subfoveal choroidal thickness.

We classified the patients into two groups based on the presence (fluid group) or absence (dry group) of retinal fluid, including SRF and/or IRF, on OCT. The fluid group was defined as eyes in which retinal fluid was observed at either the 1-year or 2-year time point after switching. The prevalence of retinal fluid was 100% at baseline, decreasing to 16.2% at one year and 21.6% at two years after switching (one year, *p* < 0.001; two years, *p* < 0.001; Figure 1A), whereas there was no significant difference in the prevalence of retinal fluid between 1 year and 2 years after switching (*p* = 0.55). The cumulative prevalence of retinal fluid at one and two years was 24.3%, and these eyes were classified as the fluid group. The treatment outcomes of the dry and fluid groups were compared with respect to BCVA, CRT, SFCT, total number of injections, and injection intervals. No significant differences were observed between the dry and fluid groups in relation to the BCVA (Figure 1B and Table 1) or SFCT (Figure 1D and Table 1) throughout the study. However, CRT (Figure 1C and Table 1) and the total number of injections (Table 1) were significantly higher, and the injection interval (Figure 1E and Table 1) was significantly shorter in the fluid group than in the dry group (CRT, *p* = 0.01; injection number, *p* < 0.01; injection interval, *p* = 0.006). No other significant differences were observed between the two groups.

The longitudinal changes in visual acuity, retinal and choroidal thickness, and injection interval were evaluated separately in the dry and fluid groups, as shown in Figure 1. In the dry group, the mean BCVA significantly improved at 1 year (*p* = 0.03) but showed no significant difference at 2 years compared to baseline (*p* = 0.17) (Figure 1B). The mean CRT significantly decreased at both 1 year (*p* < 0.001) and 2 years (*p* < 0.001) relative to the baseline (Figure 1C). Similarly, SFCT significantly decreased at 1 year (*p* < 0.001) and 2 years (*p* < 0.001) (Figure 1D). The mean injection interval was significantly extended at 1 year (*p* = 0.02) and 2 years (*p* < 0.001) compared to the baseline (Figure 1E). In the fluid group, no statistically significant changes were observed in BCVA (Figure 1B), CRT (Figure 1C), or injection interval (Figure 1E) at either 1 or 2 years compared to baseline (BCVA: *p* = 0.85; CRT: *p* = 0.24; injection interval: *p* = 0.063). The mean SFCT showed a significant decrease at 2 years (*p* = 0.03), but no significant difference at 1 year (*p* = 0.22) when compared to baseline (Figure 1D). No other significant longitudinal changes were observed within either group.

### 3.3. Clinical Characteristics of the Dry and Fluid Groups

We compared the clinical characteristics of the dry and fluid groups (Table 2). The presence of PH differed significantly between the two groups (*p* = 0.01); the prevalence of PH was 57.1% in the dry group and 11.1% in the fluid group (*p* = 0.01). No significant differences in the mean age, baseline BCVA, CRT, SFCT, follow-up period, total number of injections, and injection interval before switching were observed between the two groups. Similarly, no significant differences were observed in sex distribution, the presence of ever-smokers and polypoidal lesions, or the prevalence of IRF and SRF. Additionally, the prevalence of drusen in the fellow eye, as well as SHRM in the affected eye, did not differ significantly between the groups. However, the PH significantly differed in the dry group versus the fluid group (*p* = 0.01).

### 3.4. Injection Frequency and Retinal Fluid Status in the PH and Non-PH Groups

**Figure 2:**
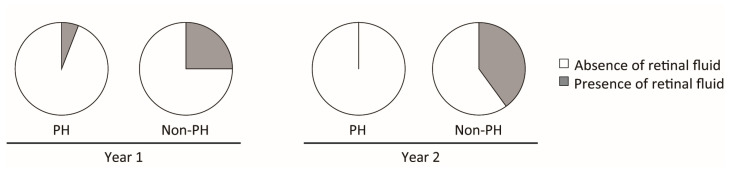
Retinal fluid status in MNV eyes of the PH and non-PH groups. The prevalence of retinal fluid at one and two years after the switch was 5.9% (1/17 eyes) and 0.0% (0/17 eyes) for the PH group and 25.0% (5/20 eyes) and 40.0% (8/20 eyes) for the non-PH group, respectively.

**Figure 3:**
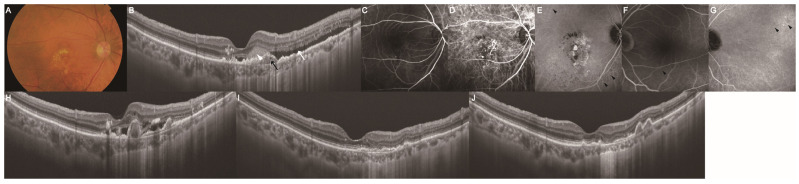
Development of MNV in a 71-year-old woman with PH in the fellow eye. Images (**A**–**F**) were obtained at the initiation of anti-VEGF therapy. Image (**G**) was obtained at the time of switching to IVBr, and images (**H**,**I**) were obtained one and two years after the switch, respectively. (**A**) Color fundus photograph showing an orange/red nodular lesion accompanied by hard exudates and hemorrhage in the macular area. (**B**) B-scan with a swept-source OCT image of the macula showed PED (black arrow) with SRF (white arrow) and SHRM (white arrowhead). (**C**) Early-phase FA showed the area of hyperfluorescence corresponding to the orange/red nodular lesion observed in the macular area. (**D**) Early-phase ICGA showed hyperfluorescence of abnormal choroidal vessels with polypoidal lesions corresponding to the orange/red nodular lesion observed in the macular area. (**E**) Late-phase ICGA showed PH (black arrowhead), which was located apart from the site of macular neovascularization. (**F**) Late-phase FA of the fellow eye showed no obvious abnormalities. (**G**) Late-phase ICGA of the fellow eye showed PH (black arrowhead) within the choroidal vascular hyperpermeability. (**H**) B-scan with a swept-source OCT image of the macula showed PED with SRF. (**I**,**J**) No SRF was observed on B-scan with a swept-source OCT image of the macula. FA, fluorescein angiography; ICGA, indocyanine green angiography; MNV, macular neovascularization; OCT, optical coherence tomography; PED, pigment epithelial detachment; PH, punctate hyperfluorescence; SHRM, subretinal hyperreflective material; SRF, subretinal fluid.

**Figure 4:**
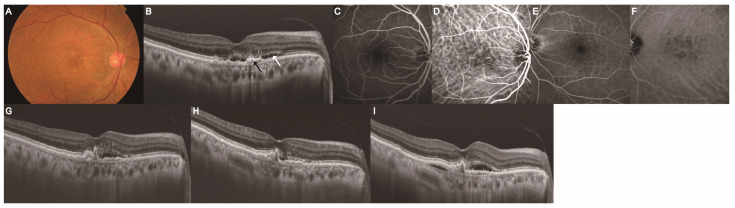
Development of MNV in a 72-year-old woman without PH in the fellow eye. Images (**A**–**F**) were obtained at the initiation of anti-VEGF therapy. Image (**G**) was obtained at the time of switching to IVBr, and images (**H**,**I**) were obtained one and two years after the switch, respectively. (**A**) Color fundus photograph showing grayish-white lesions in the macular area. (**B**) B-scan with a swept-source OCT image of the macula showed PED (black arrow) with SRF (white arrow). (**C**) Early-phase FA revealed a hyperfluorescent area on the nasal side of the macula. (**D**) Early-phase ICGA showed hyperfluorescence of abnormal choroidal vessels on the nasal side of the macula. (**E**) Late-phase FA of the fellow eye showed no obvious abnormalities. (**F**) No PH was observed on late-phase ICGA of the fellow eye. (**G**–**I**) B-scan with a swept-source OCT image of the macula showed PED with SRF. FA, fluorescein angiography; ICGA, indocyanine green angiography; MNV, macular neovascularization; OCT, optical coherence tomography; PED, pigment epithelial detachment; PH, punctate hyperfluorescence; SHRM, subretinal hyperreflective material; SRF, subretinal fluid.

The treatment outcomes of the PH and non-PH groups were compared; they included the total number of injections, injection intervals, and presence of retinal fluid (Table 3 and Figure 2). No significant differences were observed between the PH and non-PH groups in the total number of injections or injection intervals throughout the study. However, the prevalence of retinal fluid was higher two years after the switch for the non-PH group than for the PH group (*p* < 0.001). Representative cases of PH (Figure 3) and non-PH (Figure 4) are shown. In the patient described in Figure 3, who exhibited PH in the fellow eye, retinal fluid completely resolved, and the height of the pigment epithelial detachment (PED) decreased after the switch to IVBr. Conversely, in the patient without PH in the fellow eye depicted in Figure 4, retinal fluid remained despite the switch to IVBr.

## 4. Discussion

This study demonstrated that switching to IVBr for patients with nAMD who had retinal fluid despite IVA administration every 4–8 weeks resulted in reductions in retinal and choroidal thickness, extension of the injection interval, and achievement of a dry macula in approximately 80%. Furthermore, PH is suggested to be a favorable prognostic biomarker for treatment outcomes because patients with PH achieved dry macula at a significantly higher rate. To the best of our knowledge, no previous studies have evaluated the predictive factors for treatment response over two years following a switch to IVBr for patients with nAMD. Persistent retinal fluid is associated with worse visual outcomes. Therefore, patients with aflibercept-refractory nAMD who have PH may benefit from switching to IVBr therapy.

Our study revealed that switching to IVBr resulted in a 4.1-week extension of the injection interval and a dry macula in 78.4% of patients with aflibercept-refractory nAMD at two years. These results are consistent with the reports of previous studies [7,8,9]. Kikushima et al. evaluated the one-year outcomes of switching to IVBr for patients with nAMD who had residual retinal fluid despite IVA administered at intervals of eight weeks or less, reporting a 4.5-week extension of the injection interval and a dry macula rate of 43%. Kim et al. evaluated the six-month outcomes after switching to bimonthly IVBr for patients with nAMD who had residual retinal fluid despite monthly IVA injections and reported a dry macula rate of 75%. Ueda-Consolvo et al. assessed 18-month outcomes after switching to IVBr for patients with Type I MNV and polypoidal choroidal vasculopathy (PCV) who had been treated with IVA. The dry macular rate was not assessed in their study, but they reported a 4.2-week extension of the injection interval for Type I MNV and a 4.8-week extension for PCV. These findings suggest that switching to IVBr should be actively considered for patients who have resistance to IVA, as residual retinal fluid may be associated with poorer visual outcomes [16], and extension of the treatment interval can reduce the burden on both patients and healthcare providers.

In the present study, switching to IVBr led to anatomical improvements at two years after the switch; however, no significant improvement in visual acuity was observed. Kikushima et al. [7] and Ueda-Consolvo et al. [9] observed anatomical improvements without significant visual acuity gains, whereas Kim et al. [8] reported improvements in both anatomical parameters and visual acuity. This discrepancy may be attributed to irreversible photoreceptor damage and chronic structural changes caused by long-standing retinal fluid accumulation [17], which is supported by the duration of treatment before switching: 54.3 months in our study, 57.6 months in Kikushima et al., 57 months in the Type I MNV group and 59 months in the PCV group reported by Ueda-Consolvo et al., and 17 months in Kim et al. In a study examining the relationship between the duration of residual retinal fluid and visual outcomes, Kim et al. reported that a longer duration of retinal fluid absence was associated with a greater improvement in visual acuity [18]. Therefore, timely switching to IVBr should be considered for patients with residual retinal fluid treated with IVA to prevent irreversible retinal damage and preserve visual function.

Identifying biomarkers that can predict treatment response to IVBr is of critical importance, as such markers may aid in determining whether to switch from a prior anti-VEGF agent. In a previous report, Hirayama et al. demonstrated that fewer prior anti-VEGF injections and pre-switching SFCT of less than 250 µm were associated with a favorable response to IVBr [10]. Ueda-Consolvo et al. reported that smaller lesion size and lower aflibercept frequency were associated with a favorable response in patients with nAMD who switched from IVA and had Type I MNV. For PCV, smaller lesions and fewer polyps were associated with a better response [9]. Yeom et al. investigated the outcomes of switching to IVBr for patients with nAMD previously treated with bevacizumab, ranibizumab, or aflibercept and found that more previous injections, thinner baseline CRT, and the presence of prechoroidal cleft or polypoidal lesions were associated with a better response [11]. However, the previously reported predictors, including the number of prior injections, CRT, and SFCT, were not identified as predictive factors for treatment. These discrepancies may be due to differences in patient ethnicity or the type of anti-VEGF agent used before switching.

Our study identified PH as a predictive biomarker associated with a favorable response to IVBr switching. PH is a focal area of hyperfluorescence observed in mid- to late-phase ICGA and is frequently detected in both the affected and fellow eyes of patients with central serous chorioretinopathy (CSC) [13]. Subsequent studies demonstrated that PH is also commonly observed in both eyes of patients with PCV [18]. Furthermore, large PH has been reported as a clinical feature of pachydrusen [19], clearly distinguishing it from soft drusen, which shows hypofluorescence on ICGA [20]. These findings suggest that PH represents a common phenotype in the pathogenesis of pachychoroid diseases. The term “pachychoroid” refers to a disease entity that systematically encompasses macular disorders characterized by a thickened choroid with dilated choroidal vessels in Haller’s layer and attenuation of the choriocapillaris and Sattler’s layers. This spectrum includes CSC [21], pachychoroid pigment epitheliopathy (PPE) [22], pachychoroid neovasculopathy (PNV) [23], PCV [24], and peripapillary pachychoroid syndrome [25]. Anti-VEGF therapy has been reported to be more effective for patients with PNV than for those with nAMD [26,27], highlighting the importance of accurately distinguishing between PNV and nAMD. However, choroidal thickness is influenced by factors such as patient age and axial length, and there is currently no clear cutoff value for defining choroidal thickening. Consequently, there are currently no established diagnostic criteria to differentiate PNV from nAMD at present [28,29]. We previously evaluated the one-year outcomes of IVA in patients with nAMD without drusen in the fellow eye and found that patients with PH had a thicker choroid and required fewer injections [14]. In another of our studies, we assessed the one-year outcomes of PRN-based IVA in patients with nAMD whose fellow eyes had pachydrusen and reported a lower retreatment rate for this group [15]. Similarly, Fukuda et al. evaluated the one-year outcomes of PRN-based IVA for patients with PCV with pachydrusen in the fellow eye and reported a lower rate of retreatment [20]. These findings suggest that patients with nAMD and PH may have pachychoroid-driven MNV. Considering the absence of standardized diagnostic criteria for pachychoroid spectrum diseases, PH may serve as a useful biomarker for differential diagnosis.

The limitations of this study include its retrospective design and relatively small sample size, which may have affected the generalizability of the results. Another limitation is that the evaluation of PH was based on ICGA images obtained at the initiation of anti-VEGF therapy, not at the time of switching to brolucizumab. Therefore, the PH status may not precisely reflect the ocular condition immediately prior to the switching. In addition, a single physician (H.K.) proposed the initial treatment regimen at the time of switching to brolucizumab for all cases, but selection bias cannot be ruled out. Further validation using larger prospective studies is required. However, the study population was restricted to patients who had residual retinal fluid despite receiving intravitreal aflibercept (IVA) at 4- to 8-week intervals, thereby enhancing the validity of the findings in the context of identifying predictive biomarkers.

## 5. Conclusions

Switching to brolucizumab for eyes with nAMD refractory to aflibercept injections led to a dry macula rate of 80% two years after the switch, supporting IVBr as a viable treatment option. Furthermore, PH was associated with a 100% dry macula rate at two years, and it may serve as a favorable prognostic factor for the treatment response to brolucizumab.

## Figures and Tables

**Table 1:** Treatment outcomes of the dry and fluid groups.

	Dry	Fluid	*p*
Number of eyes, no. (%)	28 (75.7)	9 (24.3)	
BCVA (logMAR), mean (SD)			
Baseline	0.24 (0.26)	0.22 (0.21)	0.75
Year 1	0.18 (0.31)	0.13 (0.14)	0.83
Year 2	0.21 (0.37)	0.23 (0.34)	0.71
CRT (µm), mean (SD)			
Baseline	240.0 (61.3)	241.8 (37.6)	0.85
Year 1	190.2 (41.9)	220.0 (29.5)	0.04
Year 2	191.4 (41.0)	234.3 (36.0)	0.01
SFCT (µm), mean (SD)			
Baseline	202.8 (110.0)	163.2 (63.8)	0.44
Year 1	187.8 (110.3)	154.9 (65.5)	0.60
Year 2	170.6 (107.9)	142.0 (56.4)	0.71
Total injections (no.), mean (SD)			
Year 1	6.7 (0.9)	7.0 (0.7)	0.38
Year 2	5.0 (1.1)	6.2 (0.8)	0.005
Total	11.6 (1.7)	13.2 (1.2)	0.02
Injection interval (week), mean (SD)			
Baseline	6.7 (1.7)	5.6 (1.9)	0.11
Year 1	9.2 (1.7)	8.2 (0.7)	0.10
Year 2	11.1 (2.6)	8.7 (1.4)	0.006

BCVA, best-corrected visual acuity; CRT, central retinal thickness; SD, standard deviation; SFCT, subfoveal choroidal thickness.

**Table 2:** Clinical characteristics of the dry and fluid groups.

	Dry	Fluid	*p*
Number of eyes, no. (%)	28 (75.7)	9 (24.3)	
Age (years), mean (SD)	72.2 (7.7)	71.0 (5.2)	0.97
Sex (female), No. (%)	13 (46.4)	2 (22.2)	0.19
Smoking habits (ever-smokers), No. (%)	19 (67.9)	5 (55.6)	0.51
Presence of IRF, No. (%)	5 (17.9)	1 (11.1)	0.62
Presence of SRF, No. (%)	25 (89.3)	9 (100.0)	0.19
Presence of polypoidal lesion, No. (%)	13 (46.4)	5 (55.6)	0.63
Presence of PH, No. (%)	16 (57.1)	1 (11.1)	0.01
Presence of drusen in the fellow eye, No. (%)			0.25
Soft drusen	9 (32.1)	2 (22.2)	
SDD	1 (3.6)	1 (11.1)	
Pachydrusen	5 (17.9)	0 (0.0)	
No drusen	13 (46.4)	6 (66.7)	
Presence of SHRM in the affected eye, No. (%)			0.06
Exudation	3 (10.7)	0 (0.0)	
Hemorrhage	3 (10.7)	4 (44.4)	
No SHRM	22 (78.6)	5 (55.6)	
Follow-up duration (mo), mean (SD)	52.4 (46.6)	60.0 (48.4)	0.76
Total injections (no.), mean (SD)	24.6 (23.3)	34.2 (30.3)	0.35
Injection interval (week), mean (SD)	6.7 (1.7)	5.6 (1.9)	0.11
Baseline, mean (SD)			
BCVA (logMAR)	0.24 (0.26)	0.22 (0.21)	0.75
CRT (µm)	240.0 (61.3)	241.8 (37.6)	0.85
SFCT (µm)	202.8 (110.0)	163.2 (63.8)	0.44

BCVA, best-corrected visual acuity; CRT, central retinal thickness; IRF, intraretinal fluid; PH, punctate hyperfluorescence; SD, standard deviation; SDD, subretinal drusenoid deposit; SFCT, subfoveal choroidal thickness; SHRM, subretinal hyperreflective material; SRF, subretinal fluid.

**Table 3:** Treatment outcomes of the PH and non-PH groups.

	PH	Non-PH	*p*
Number of eyes, no. (%)	17 (45.9)	20 (54.1)	
Total injections (no.), mean (SD)			
Baseline	6.6 (1.1)	6.9 (0.7)	0.48
Year 1	4.9 (1.1)	5.6 (1.1)	0.07
Year 2	11.5 (1.7)	12.5 (1.6)	0.11
Injection interval (week), mean (SD)			
Baseline	6.6 (1.5)	6.3 (2.0)	0.78
Year 1	9.6 (1.8)	8.4 (1.0)	0.01
Year 2	11.1 (2.7)	10.0 (2.4)	0.18
Presence of Retinal Fluid, No. (%)			
Year 1	1 (5.9)	5 (25.0)	0.10
Year 2	0 (0.0)	8 (40.0)	<0.001

PH, punctate hyperfluorescence; SD, standard deviation.

## Data Availability

The data used to support the findings of this study are restricted by the Kawasaki Medical School Ethics Committee to protect patient privacy. Data are available from Hiroyuki Kamao (hironeri@med.kawasaki-m.ac.jp) for researchers who meet the criteria for access to confidential data.
